# Spinorphin Molecules as Opportunities for Incorporation into Spinorphin@AuNPs Conjugate Systems for Potential Sustained Targeted Delivery to the Brain

**DOI:** 10.3390/ph18010053

**Published:** 2025-01-05

**Authors:** Stela Georgieva, Petar Todorov, Jana Tchekalarova

**Affiliations:** 1 Department of Analytical Chemistry, University of Chemical Technology and Metallurgy, 1756 Sofia, Bulgaria; 2 Department of Organic Chemistry, University of Chemical Technology and Metallurgy, 1756 Sofia, Bulgaria; pepi_37@abv.bg; 3 Institute of Neurobiology, Bulgarian Academy of Sciences, 1113 Sofia, Bulgaria; janetchekalarova@gmail.com

**Keywords:** nanoparticles, AuNPs, carriers, drug delivery systems, peptide, spinorphin, anticonvulsant activity

## Abstract

**Background:** This study explores the potential for the synthesis of peptide nanosystems comprising spinorphin molecules (with rhodamine moiety: Rh-S, Rh-S5, and Rh-S6) conjugated with nanoparticles (AuNPs), specifically peptide Rh-S@AuNPs, peptide Rh-S5@AuNPs, and peptide Rh-S6@AuNPs, alongside a comparative analysis of the biological activities of free and conjugated peptides. The examination of the microstructural characteristics of the obtained peptide systems and their physicochemical properties constitutes a key focus of this study. **Methods:** Zeta (ζ) potential, Fourier transformation infrared (FTIR) spectroscopy, circular dichroism (CD), scanning electron microscopy (SEM-EDS), transmission electron microscopy (TEM), and UV–Vis spectrophotometry were employed to elucidate the structure–activity correlations of the peptide@nano AuNP systems. **Results:** The zeta potential values for all the Rh-S@AuNPs demonstrate that the samples are electrically stable and resistant to flocculation and coagulation. The absorption of energy quanta from UV–Vis radiation by the novel nanopeptide systems does not substantially influence the distinctive signal of AuNPs, which is situated at around 531 nm. The FTIR measurements indicate the signals associated with the unique functional groups of the peptides, whereas circular dichroism verifies the synthesis of the conjugated nanocomposites of the spinorphin@AuNP type. An analysis of the SEM and TEM data revealed that most AuNPs have a spherical morphology, with an average diameter of around 21.92 ± 6.89 nm. The results of the in vivo studies showed promising findings regarding the anticonvulsant properties of the nanocompounds, especially the Rh-S@AuNP formulation. **Conclusions**: All the nanocompounds tested demonstrated the ability to reduce generalized tonic–clonic seizures. This suggests that these formulations may effectively target the underlying neuronal hyperexcitability. In addition, the prepared Rh-S@AuNP formulations also showed anticonvulsant activity in the maximal electroshock test performed in mice, which was evident after systemic (intraperitoneal) administration. The study’s findings indicate that conjugates can be synthesized via a straightforward process, rendering them potential therapeutic agents with biological activity.

## 1. Introduction

Nanoparticles can be applied in many fields, such as healthcare and medicine, information and communication technologies, energy, and the environment [1,2,3,4]. They can be utilized as treatments or as carriers for therapeutic compounds, depending on their chemical and physical characteristics [5]. There are several kinds of nanoparticles [6]; gold nanoparticles (AuNP) can be readily functionalized with a variety of biomolecules (e.g., medicines, antibodies, peptides, or targeting ligands) to improve their targeting capabilities [7,8,9]. These types of particles are preferred because gold is generally non-toxic, making AuNP suitable for biomedical applications, especially when modified to minimize toxicity and the immune response. The basic mechanisms of incorporation and the actions of gold nanoparticles in biological applications have been also known [1,2,3,4,5,6,7,8]. It has been established that AuNPs themselves attack tumor tissues and can accumulate in them due to the permeable vasculature of tumors and their poor lymphatic drainage, a phenomenon known as the EPR effect [5,10]. This allows for more selective accumulation in tumors compared to healthy tissues. By functionalizing AuNPs with specific molecules (e.g., small peptide molecules) that recognize the overexpressed receptors on the surface of target cells (such as cancer cells), AuNP can be actively targeted and deliver drugs directly to the diseased tissue, improving therapeutic efficacy and reducing the off-target effects [5]. Moreover, AuNPs serve as excellent carriers for various types of therapeutic agents, including chemotherapeutics, siRNA, DNA, and proteins, due to their high surface area and ease of drug attachment [5]. On the other hand, a controlled release of the target drug molecule from AuNPs is observed through various changes in the environment such as changes in pH, temperature, or the use of external stimulation like light or magnetic fields [11]. In the literature, there are data for AuNPs as carriers of antibiotics and their potential to overcome issues related to antibiotic resistance [12,13].

**Scheme 1:**
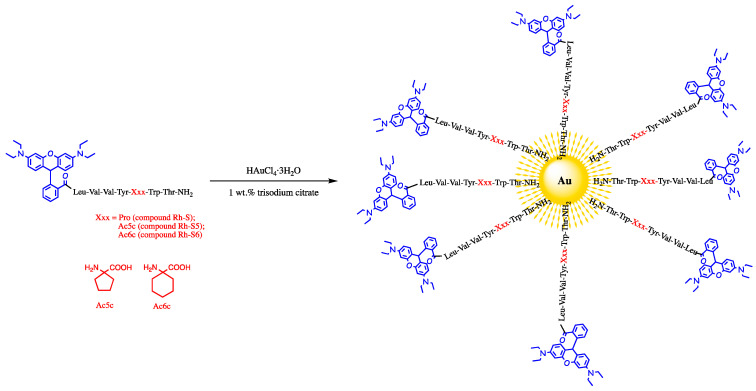
Schematic presentation of the synthesis of rhodamine spinorphin peptides conjugated with gold nanoparticles.

Proteins and peptides have been used as medicinal agents much more frequently in recent years [14,15,16]. Sadly, proteins can be digested by enzymes and, in addition to their therapeutic benefits, can trigger an unintended immunological reaction [17]. The dosage of the medication required to provide the desired therapeutic effect can be decreased and unwanted side effects can be minimized by using drug carriers [17]. Currently, most clinical treatments are highly invasive, highly destructive, and create numerous unwanted side effects. In this regard, based on the presented manuscript, the idea of obtaining and studying the properties of biologically active peptide analogs and preparing nanopeptide systems for biological testing with achievable effects is presented. A growing number of disorders, including cancer, diabetes mellitus, hypertension, epilepsy, and chronic pain, are being treated using peptides [18]. Blocking the enzymes involved in the conversion of angiotensin peptides requires a search for substances with more effective anti-inflammatory and analgesic activity and without toxicity. Therefore, chemists and pharmacologists are interested in creating novel analogues of blood protein peptides, such as spinorphin derivatives that contain a variety of non-proteinogenic conformationally constrained amino acids, unnatural amino acids, phenytoin, and/or rhodamine residues with neuro-biological activity. Furthermore, fluorophores can be included in peptide scaffolds’ structures while retaining biological activity, expanding the potential for a fluorescence sensor design and its uses in biochemistry and medicine [19,20]. These fluorophores are also useful labels for peptides and proteins, for visualizing these biomolecules inside cells [21,22]. Currently, the information from research on the combination of nanoparticles and peptides is extremely limited. The swift convergence and growth of the nanoparticle and peptide shuttle fields have both led to a fundamental understanding of certain diseases and offer immense potential for combining transport with therapies [23,24,25]. Depending on the scientific approach, various strategies for the delivery of nanomedicines have been developed [26]. Unfortunately, there is currently a gap in understanding both the mechanisms of nanoparticle clearance from tissues and their long-term effects, and the clinical application of these nanoconstructs does not hide the fact that this requires higher levels of research. Since nanoparticles are taken up by cells more efficiently than larger macromolecules, they are an excellent material for a delivery system, which is why the direction of therapeutic research is focused on the combination of nanomaterials with targeting components [26]. Using a peptide delivery mechanism would enable a quicker distribution to the location of action, longer bodily retention, and fewer adverse effects. Additionally, it can raise the physiologically active molecule’s concentration in the target tissues and shield it from quick elimination [27]. As a result, the use of proteins as therapeutic agents has increased recently, in addition to the development of small molecules. Because of their greater activity, lesser toxicity, and specificity of action in comparison to traditional medications, they hold significant promise [27,28]. As previously stated, a significant obstacle to efficient and targeted delivery is that the proteins that are delivered into the body are susceptible to enzymatic degradation, have a short half-life, can elicit an immune response because of their complex structure, and are poorly permeable through biological membranes [28,29]. Active transport to the location is made possible by the use of carriers, which protect peptides and proteins from environmental factors while preserving their stability and lowering the immune response. This method frequently improves enzymatic activity and biocompatibility. Therefore, the scientific objectives of this manuscript encompass the presentation of new biologically active peptide analogs with a fluorophore for the preparation of nanopeptide systems through the use of metal nanoscale carriers for peptides and a comparative study of the biological properties of free peptides with those referred to as molecules on nanocarriers (see Scheme 1). According to the objective, we choose to synthesize and characterize the derivatives of endogenous opioid peptides (spinorphin derivatives), as the pharmacological effects of opioids are mediated through the binding and activation of membrane-bound opioid receptors located in the central and peripheral nervous systems [30]. Conjugating the peptide with nanoparticles has the potential to target the brain with minimal degradation and toxicity. The significant drawbacks of well-known peptide ligands, including their relative incapacity to pass across the blood–brain barrier, their long-term toxicity and poor biostability, their numerous adverse effects, and more, may be addressed by novel approaches to drug creation.

## 2. Results and Discussion 

### 2.1. Chemistry

Preparative quantities of the peptide biomolecules rhodamineB-Leu-Val-Val-Tyr-Pro-Trp-Thr-NH_2_ (Rh-S), rhodamineB-Leu-Val-Val-Tyr-Ac5c-Trp-Thr-NH_2_ (Rh-S5), and rhodamineB-Leu-Val-Val-Tyr-Ac6c-Trp-Thr-NH_2_ (Rh-S6), where Ac5c is 1-aminocyclopentanecarboxylic acid and Ac6c is 1-aminocyclohexane carboxylic acid, were obtained by manual solid-phase peptide synthesis, according to the Fmoc-strategy described in detail by our group [31]. The approach outlined by Dong et al. was used to create the newly synthesized spinorphin analogs conjugated with the gold nanoparticles [32]. Scheme 1 shows the synthesis of the three new nanopeptide systems, Rh-S@AuNPs, Rh-S5@AuNPs, and Rh-S6@AuNPs. The attachment of a peptide to a nanoparticle by different methods has been reported in the literature. Chemical conjugation serves as a robust method for creating peptide nanosystems involving the covalent attachment of peptides to nanoparticles [33]. Three functional groups in the peptide molecules predominantly utilized for the synthesis of these conjugates are carboxyl groups (–COOH), amine groups (–NH_2_), and thiol groups (–SH). Amino groups are present in the side chains of lysine residues or at the N-terminus of peptides [33]. Their positive charge at a physiological pH often positions them on the surface, facilitating interactions. Of the listed functional groups, the tested peptides contain an amide group at the C-terminus, which gives us reason to assume that the conjugation occurs precisely through the interaction of this group with the gold nanoparticle, as depicted in Scheme 1.

### 2.2. Spectral Characterization

**Figure 1:**
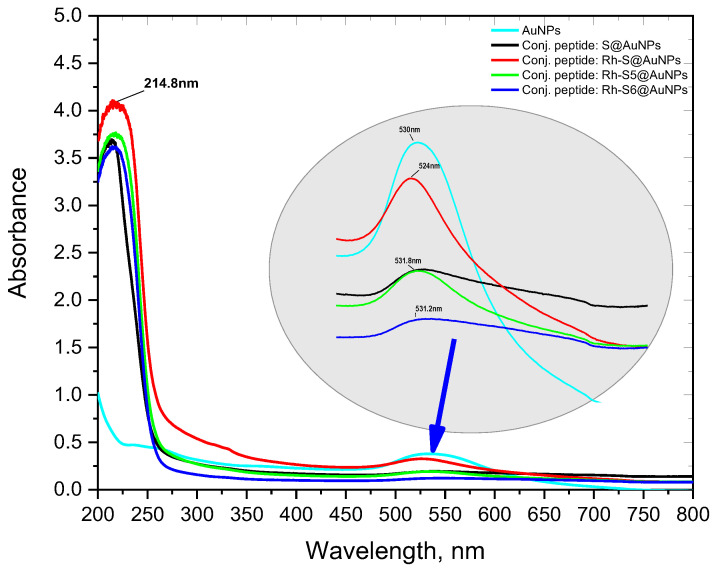
UV–Vis spectrum of AuNPs and Peptide@AuNP conjugate.

UV–Vis spectrophotometry was used to characterize the interactions with electromagnetic radiation of the pure nanoparticles and the resulting nanopeptide systems (Figure 1). As can be seen from the figure, a characteristic band for gold nanoparticles is observed at wavelengths around 531 nm. As a result of directing the peptide molecules to the surface of the metal nanoparticle and the subsequent conjugation with it, a decrease in the intensity of the spectral line at 531 nm is observed without changing its position. This change can be attributed to the adsorption of the peptide on the flat metallic gold surface as a result of the resonant oscillation of electrons at the boundary of the nanopeptide system (material) with positive and negative dielectric constants, caused by the incoming light, also known as surface photoresonance (SPR). These results show that the spinorphin@AuNP systems retain their photostability despite conjugation to the gold nanosurface. The shapes and sizes of the gold nanoparticles, AuNPs, used are shown in the TEM images. However, it was found that the size or shape of the spinorphin conjugates has little effect on their photostability (the signal at 531 nm, Figure 1). As can be seen, the longer the peptide scaffold, the lower the intensity of the observed band. This may be due to structural interference when the peptide “wraps” on the nanosurface which leads to more difficult access for energy quanta to the metal part of the nanopeptide system. At the same time, the recorded absorption signals at about 220 nm, corresponding to the peptide bonds present [34] and the chromophore groups in tyrosine and tryptophan, do not change their intensity or location relative to those of pure peptides [35]. The presence of a peptide bond is evidenced by the highly intense stripes at 200–230 nm. Basically, the π→π∗ transitions of the >C=O in the peptide bonds occurred through UV absorption of the peptide molecule in the range of 180 to 230 nm. The aromatic side-chains of the indole of Trp, the phenol rings of Tyr, and rhodamine are primarily responsible for the absorption of the π-electron systems of the aromatic groups in the range of ≈300 nm [36,37].

**Figure 2:**
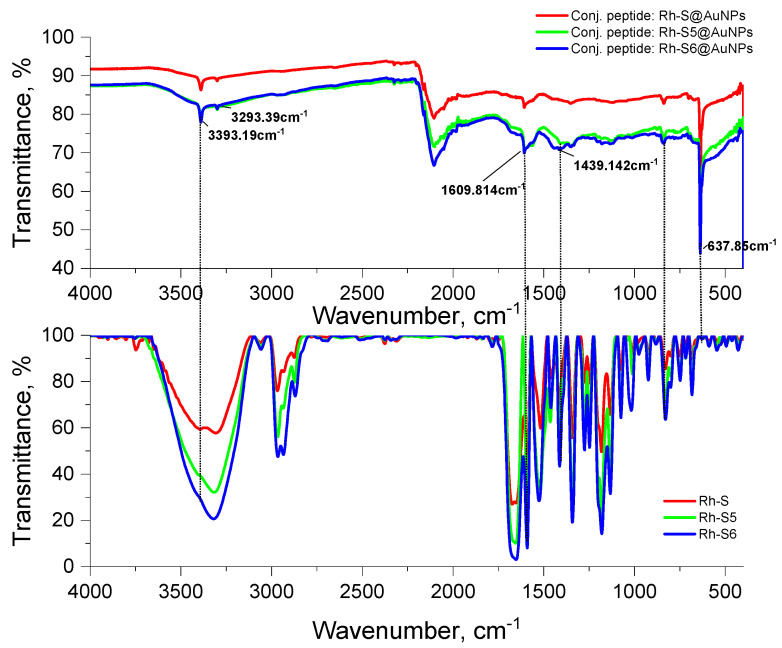
FTIR spectrum of AuNPs and Peptide@AuNP conjugate.

FTIR was used to detect the participation of the different functional groups present in the nanopeptide system. As is known, the characteristic IR lines corresponding to amide I (1600–1700 cm^−1^), amide II (1500–1600 cm^−1^), and amide III (1200–1300 cm^−1^) can be observed in the molecule of a peptide [38]. The band of amide I is mainly associated with the tensile vibration of C=O in the peptide bond combined with C-N stretching. The amide III band includes the NH in the bending plane and the C-N tensile vibrations. For a more visual representation of the effect of conjugation on the absorption IR bands, the spectra of pure peptide molecules were used for a comparison (Figure 2). The characteristic lines that are also observed in pure peptides are as follows: ~3330–3352—N-H stretching vibration (νNH), ~1701 (s)—NCO (amide) stretching, 1655–1660 cm^–1^—with a high-intensity peak of νC=O, and 1511–1439 cm^–1^ (δNH) (Figure 2). It can be said that these lines correlate with a beta sheet peptide structure rather than with an a-helix. As seen here, the absorption of radiation from the longer wavelength region by the spinorfin@nano systems results in a change in the spectral band intensity without a significant displacement observed (Figure 2). The wider peak, also observed in the conducted peptides, localized at ~3390 cm^−1^ is attributed to the N-H stretching vibration (νNH) oscillation region. Two more peaks were found at 1609 cm^−1^ and 2045 cm^−1^. The signal at 1609 cm^−1^ corresponds to amide I, as well as the stretching vibrations of the C=O (70–85%) and C-N groups (10–20%). This band strongly indicates the presence of an antiparallel beta-structure in the peptides. The peak at 2045 cm^−1^ corresponds to C–C stretching from the alkene, the bonds present in the peptide chains. The absorption band at 637 cm^−1^ was identified as deformation oscillations of N-H [38]. As a result of the obtained spectra, it can be concluded that in the studied peptide nanosystems, stripes corresponding to non-aggregated molecules and proving the preservation of the conformational structure of the conjugated peptide are detected. The resulting absorption bands of the three peptides conjugated to the metal nanoparticles visibly decrease in intensity compared to the initial “free” peptides, which could reflect the formation of the significant amounts and sizes of porosity on the surface of the peptides, as discussed in the next section.

### 2.3. Analyses by Circular Dichroism

**Figure 3:**
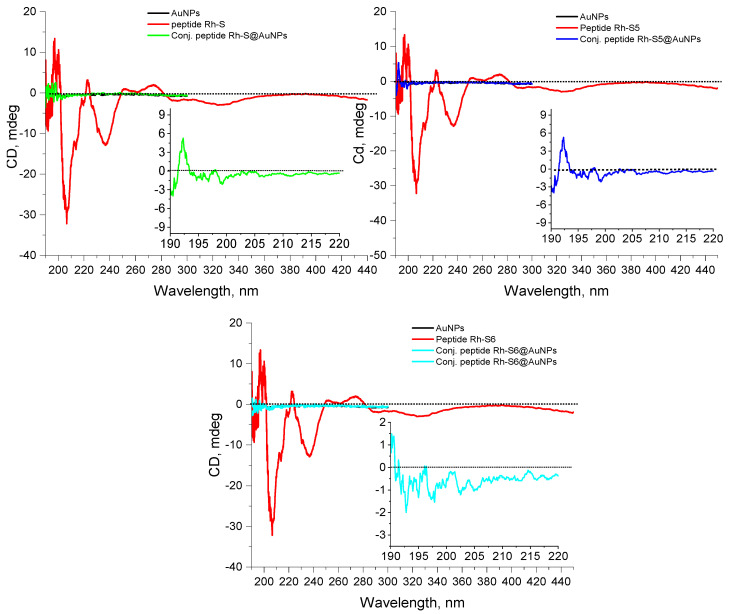
CD spectra between 190 and 440 nm of the studied peptide derivatives conjugated with a hydantoin moiety.

Like other forms of absorption spectroscopy (UV/Vis, IR, etc.), CD is particularly powerful in observing conformational changes, especially those affected after conjugation [39,40,41]. The circular dichroism phenomenon is very sensitive to the secondary structure of polypeptides and proteins (Figure 3). It has been shown that the CD spectra between 260 and approximately 180 nm can be analyzed for the different secondary structural types: an alpha helix, parallel and antiparallel beta sheets, a turn, and others. In the region of 230–178 nm, the effects of conformational changes in the spine are expected to be observed, while the CD effects at longer wavelengths (>230 nm) should isolate aromatic chromophore control [40]. In order to confirm the conformational stability of the nanopeptide conjugates, their spectra were recorded when interacting with circularly polarized light (Figure 3). It can be seen that differently intense bands are produced depending on the type of conjugate obtained. The proven content of the conformational isomers of the β-sheet and the lower content of the β-turn are seen in the large absorption bands in the negative region at ~197 nm for both the parent peptide compounds and their conjugates. This shows that the CD spectra do not contradict the conclusions drawn from FTIR spectroscopy. It can be concluded that both with irradiation with polarized light and with the spectra taken subsequently in the obtained nanopeptide systems, the integrity of the peptide molecule is preserved, while its conformational state is preserved.

### 2.4. Transmission Electron Microscopy (TEM)

**Figure 4:**
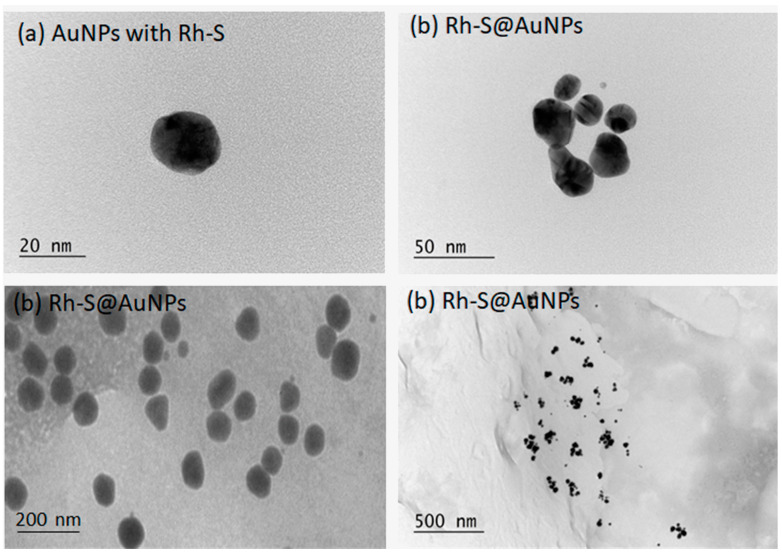
TEM images of (**a**) AuNPs with Rh-S and (**b**) Rh-S@AuNPs at different resolutions.

The size and shape of the gold nanoparticles can be adjusted to optimize cellular uptake and bio-distribution. The nanoparticles themselves can be of different shapes and sizes [41]. The TEM image of AuNPs shown in Figure 4 shows that predominantly spherical and cylindrical gold nanoparticles with an average particle diameter of 21.92 ± 6.89 nm have been formed, which are well dispersed in the reaction medium without any visible agglomeration. Typically, nanoparticles in the range of 1–100 nm are most effective for drug delivery. The resulting type (shape and size) of the AuNPs of the nanoparticles presented in this study are in good agreement with those reported by others [32,42,43,44]. Figure 4 presents TEM images of Rh-S@AuNPs at different resolutions and irradiation voltages. Identical TEM morphologies were obtained in the other conjugates corresponding to the similar structures observed by Casciaro et al. [45] and Sagar et al. [46]. It is clearly seen that a “glowing white halo” is formed around a metallic gold nanoparticle, which can be attributed to the uniform arrangement of the peptide molecule on the surface of the AuNPs. The large mobility of the size of the AuNP nanomaterial would allow the peptides to reach their targets quickly through an eventual seamless passage through the blood cerebral barriers.

### 2.5. SEM-EDS Analysis

**Figure 5:**
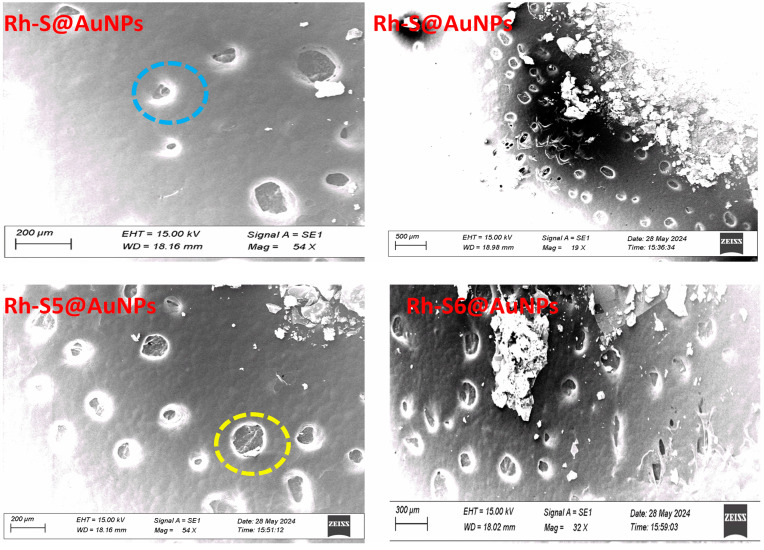
SEM images of nanopeptide system: Rh-S@AuNPs (at 200 and 500 µm), Rh-S5@AuNPs (200 µm), and Rh-S6@AuNPs (200 µm).

**Figure 6:**
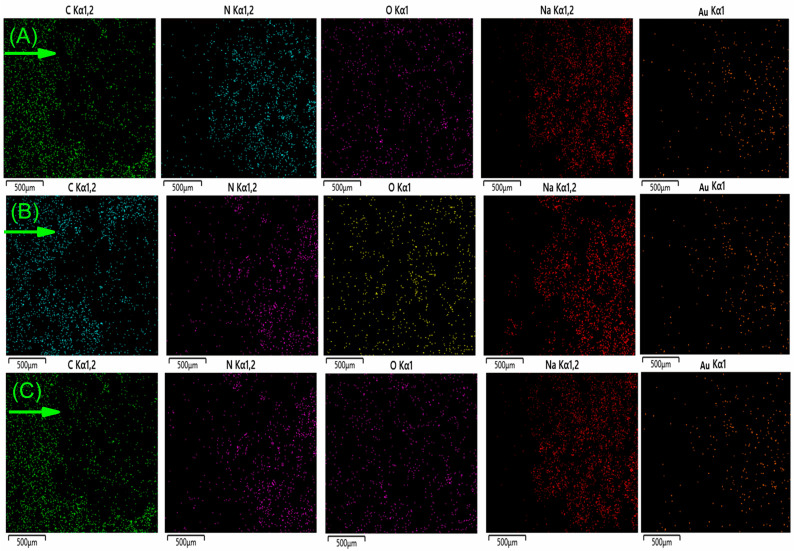
SEM images of the distribution of main components in the (**A**) Rh-S@AuNPs, (**B**) Rh-S5@AuNPs, and (**C**) Rh-S6@AuNPs.

SEM (scanning electron microscopy) peptide morphology on gold nanoparticles (AuNP) typically reveals a detailed high-resolution image of how peptides interact with or adhere to the surface of gold nanoparticles. The SEM-EDS images of Rh-S@AuNPs, Rh-S5@AuNPs, and Rh-S6@AuNPs are given in Figure 5. The images shown correspond to similar structures reported in the literature [47]. It can be seen that the porosity of the compounds is preserved, similar to that reported for the starting peptides [31]. An elemental microanalysis with respect to the atomic distribution in the samples examined the following: Rh-S@AuNPs, Rh-S5@AuNPs, and Rh-S6@AuNPs. The analysis was performed on the surface of the samples based on energy-dispersive X-ray spectroscopy (EDS) (Figure 6). The results presented show the distribution of Au, C, O, and N on the surface of the samples. A small percentage of Na and Cl content was observed as a result of the materials used in the synthesis of the nanoparticles, namely a solution of chloride trihydrate (HAuCl_4_·3H_2_O) and trisodium citrate (Na_3_C_6_H_5_O_7_), which was used in the synthesis of the AuNPs (Figure 6). The quantitative percentage of chlorine calculated with SEM-EDS is within the error limits of the method applied, which is why this distribution is not present as an image.

#### Determination of Zeta Potential

Electrophoretic light scattering (ELS), associated with measuring the speed of particles in an electric field, was used to calculate the zeta potential of the solutions of the nanoparticles conjugated to spinorphin peptides. The zeta potential (ζ) of a solution of the nanopeptide system refers to the determination of the electrical potential on the surface of particles (nanoparticles with conjugated peptide) when they are dispersed in a liquid medium. It plays a critical role in determining the stability, aggregation, and interactions of the resulting nanoparticles or colloidal systems. In drug delivery systems, a high zeta potential (positive or negative) is often desired to ensure that nanopeptides remain well dispersed in the body and to avoid a possible aggregation that may affect the drug release profile or bio-distribution. The size and sign of the zeta potential can provide an idea of the stability of the colloidal solution. The magnitude of the zeta potential indicates the degree of electrostatic repulsion between similar particles in dispersion. For very small molecules or particles, their large zeta potential plays a role in preventing aggregation. Therefore, colloids with a high zeta potential (negative or positive) are electrically stable, while colloids with a low zeta potential are prone to coagulation or flocculation. In the nano spinorphin particles studied, the resulting zeta potential value of the Rh-S@AMPs increases significantly when they are associated with AuNPs. The zeta potentials of the resulting materials range from |−38.4 ± 0.3| for Rh-S@AMPs to |−35.2 ± 0.2| mV for Rh-S5@AMPs and |−37.2 ± 0.2| mV for Rh-S6@AMPs. Negative values for the zeta potential indicate that the surface charge (the peptide has been modified to be an anion) is negative. The magnitudes of these zeta potentials are in good agreement with the findings of Sagar et al. [46], who observed that some peptides with similar structures have a negative zeta potential. A high zeta potential (in absolute terms) usually indicates that the nanopeptide systems are sufficiently charged and will repel each other, resulting in stable dispersion and the prevention of aggregation. According to the literature, those nanopeptide solutions with a zeta potential value of more than ±30 mV are considered stable [48]. According to these findings, the Rh-S@AuNPS(2), Rh-S5@AuNPs, and Rh-S6@AuNPs used in this study are resistant to coagulation and flocculation and are electrically stable.

### 2.6. Anticonvulsant Activity

**Figure 7:**
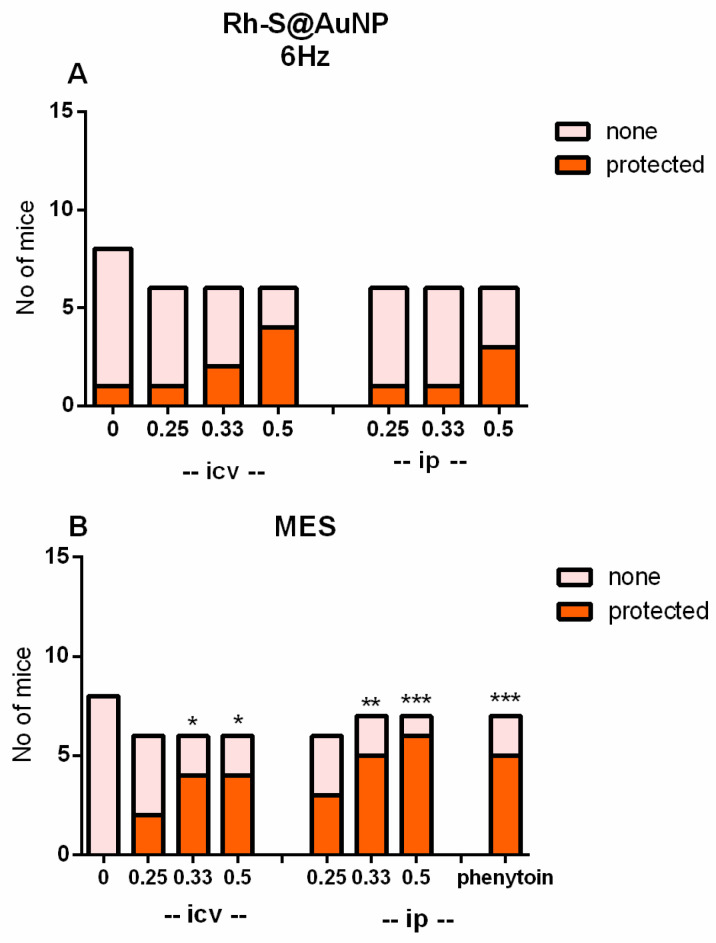
The anticonvulsant activity of Rh-S@AuNP, administered either i.c.v. or i.p. at doses of 0.25, 0.33, and 0.5 mg/kg, respectively, in the 6-Hz test (**A**) and the MES test (**B**) in mice. Phenytoin was used as a referent drug. Fisher exact test: * *p* = 0.011, ** *p* = 0.005, and *** *p* < 0.001 (Rh-S@AuNP 0.5 mg/kg i.p. and phenytoin vs. controls).

**Figure 8:**
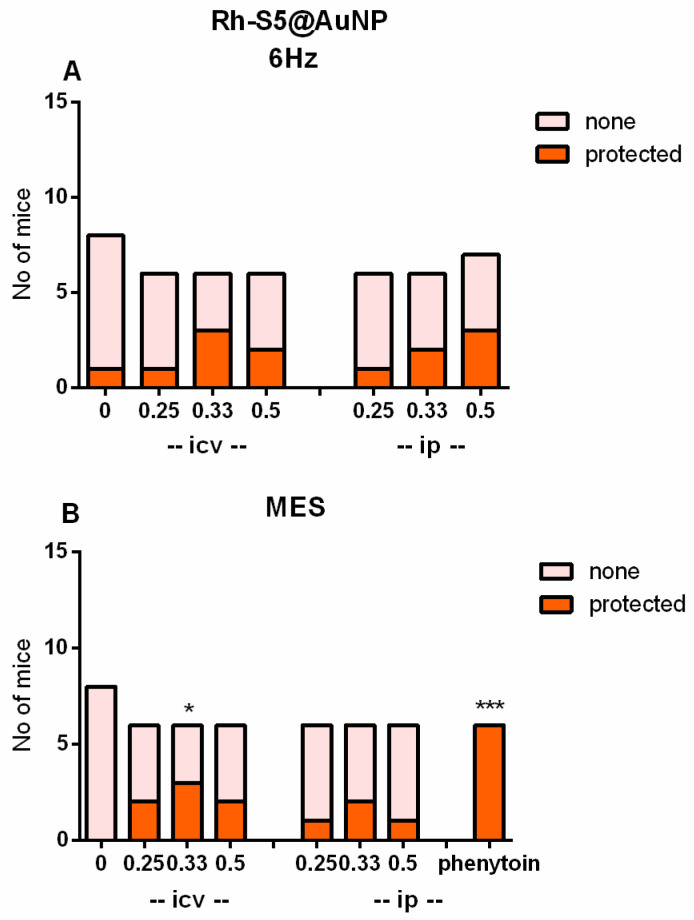
The anticonvulsant activity of Rh-S5@AuNP, administered either i.c.v. or i.p. at doses of 0.25, 0.33, and 0.5 mg/kg, respectively in the 6-Hz test (**A**) and the MES test (**B**) in mice. Phenytoin is used as a positive control. Fisher exact test: * *p* = 0.05 (Rh-S5@AuNP 0.33 mg/kg, i.c.v., vs. control), and *** *p* < 0.001 (phenytoin vs. control).

**Figure 9:**
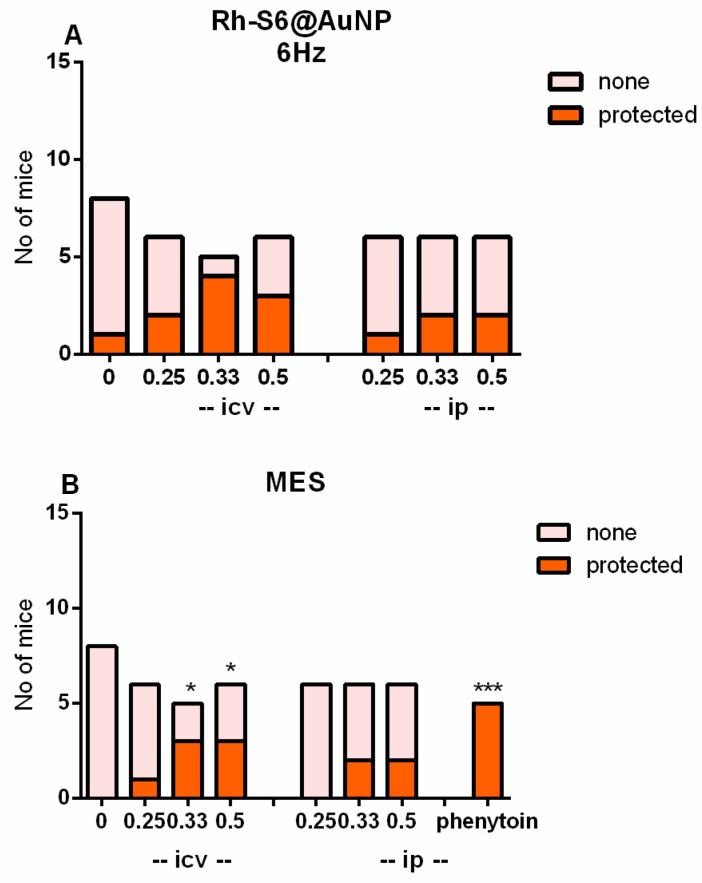
The anticonvulsant activity of Rh-S6@AuNP, administered either i.c.v. or i.p. at doses of 0.25, 0.33, and 0.5 mg/kg, respectively in the 6-Hz test (**A**) and the MES test (**B**) in mice. Phenytoin (30 mg/kg) was used as a referent drug. Fisher exact test: * *p* = 0.035 (Rh-S6@AuNP, 0.33 and 0.5 mg/kg, vs. control), and *** *p* < 0.001 (phenytoin vs. control).

Two seizure tests, a 6-Hz test and a maximal electroshock seizure (MES) test, were used to evaluate the anti-seizure activity of novel peptide analogues according the ADD protocol [49]. A referent drug, phenytoin, was applied. The results are demonstrated in Figure 7A,B, Figure 8A,B and Figure 9A,B. The drugs with anti-seizure activity in the 6-Hz test are assumed to have potential against drug-resistant epilepsy and a probable mechanism related to the blockade of fast sodium channels [49]. However, unlike the recently reported hybrid spinorphin peptide analogs (Rh-S, Rh-S5, and Rh-S6), which showed efficacy in alleviating drug-resistant 6-Hz-induced psychomotor seizures [35], none of the new formulations, Rh-S@AuNP, Rh-S5@AuNP, and Rh-S6@AuNP, were effective against psychomotor seizures in both modes of administration (i.c.v. and i.p.) (Figure 7A, Figure 8A and Figure 9A).

In the MES test, the Rh-S@AuNP showed 67% protection at doses of 0.33 and 0.5 mg/kg, respectively, after an i.c.v. infusion (*p* = 0.011 vs. the control) (Figure 7B). Furthermore, the seizure spread was suppressed with 71% (1:3) and 86% (0.5 mg/kg) efficacy, respectively, after systemic administration (*p* = 0.005 and *p* < 0.001 vs. the control). Moreover, compared to the recently reported hybrid spinorphin peptide analogs (Rh-S, Rh-S5, and Rh-S6), which were inactive in the MES test after an i.p. injection, after systemic treatment, the Rh-S@AuNP had a strong ability to suppress generalized tonic–clonic seizures, indicating that it could cross the blood–brain barrier more easily than the Rh-S–peptide complex. This effect was comparable to the referent drug phenytoin (*p* < 0.001 vs. the control).

The protection and mortality rate percentages of the MES-induced hind limb tonic seizures for the Rh-S and Rh-S@AuNP compounds, administered in three doses, i.p., are shown in Table 1.

Both the Rh-S5@AuNP compound and Rh-S6@AuNP compound showed efficacy in suppressing tonic–clonic seizures only after an i.c.v. infusion (with 0.33 mg/kg and *p* = 0.05 vs. the control (Figure 8B) and *p* = 0.035, 0.33, and 0.5 mg/kg vs. the control, respectively), but were unable to suppress seizure spread after an i.p. injection (Figure 9B). Nevertheless, their effect was weaker compared to the referent drug phenytoin (*p* < 0.001 vs. the control) (Figure 9B) in the MES test.

## 3. Materials and Methods

### 3.1. Preparation of Nanopeptide Systems of the Type Rh-S (Spinorphin Derivatives)@AuNPs

#### 3.1.1. Synthesis of AuNPs

The gold nanoparticles (AuNPs) were synthesized according to a known methodology given in the literature [32]. Au(III) chloride trihydrate (HAuCl_4_·3H_2_O, Sigma-Aldrich, St. Louis, MO, USA) was used as the starting material and the subsequent citrate reduction of Au(III) chloride with trisodium citrate was performed (Na_3_C_6_H_5_O_7_, Sigma-Aldrich). The reduction was performed at hot temperatures by adding 5 mL of 1 wt.% trisodium citrate to the gold salt solution (0.0100 mol L^−1^) with continuous stirring. After the solution acquired a red color (evidence of the reduction of Au(III) to Au°), it was allowed to cool.

#### 3.1.2. Synthesis of Spinorphin@AuNP Conjugates

After obtaining preparative amounts of the peptide derivatives by solid-phase peptide synthesis [31], the solutions with a peptide concentration were prepared as follows: 1.906 × 10^−3^ mol L^−1^ Rh-S, 1.405 × 10^−3^ mol L^−1^ Rh-S5, and 1.591 × 10^−3^ mol L^−1^ Rh-S6. To 5 mL of the respective solutions, 1 mL of the prepared AuNPs solution was added at a temperature of 400 C. After cooling the mixtures obtained, centrifugation was performed for 5 min at 250 rpm. Then, the obtained samples were examined for zeta potential, UV–Vis, FTIR, XRD, SEM, TEM, and a manifestation of biological (anticonvulsant) activity.

### 3.2. Zeta Potential

Zeta potential was estimated using a Zetasizer Nano ZS (Malvern Instruments, Malvern, UK). A total of 100 µL of each spinorphin@AuNp solution was diluted to 0.50 mL for signal obtaining. Zeta potential values were obtained as averages of 3 replicates of each sample. Smoluchowski’s approximation was used to calculate zeta potential from electrophoretic mobility.

### 3.3. Spectral Measurement

A Thermo Nicolet iS50 infrared spectrometer was used to record the ATR-IR spectra of the structures of the nanopeptide systems in the mid-infrared region of 4000–600 cm^−1^. For this purpose, a diamond crystal ATR accessory was used at a resolution of 2 cm^−1^ and 64 scans were performed. In the region of 200–800 nm (UV–Vis region), the absorption spectra of the studied systems were also recorded and measured using a Varian-Cary spectrophotometer (quartz glass cuvettes with an internal path length of 1 cm). The circular dichroism spectra were recorded using a polarized radiation, measuring the apparatus after the irradiation of the studied samples using a Jasco J-1500 CD spectrometer (resolution of 1 nm; Tokyo, Japan) in the spectral range of 160–800 nm and a constant nitrogen flow rate of 4–5 L per minute.

### 3.4. Analyses for Determining the Structural Morphology of Spinorphin@AuNP Conjugates

The morphology of the AuNPs and the spinorphin@AuNPs were examined using scanning electron microscopy (SEM) (FESEM Thermo Scientific Quattro S equipped with an EDS detector (Waltham, MA, USA) operating at 30 kV and with magnification up to 2,000,000×). Liquid samples of AuNPs and LL-37@AuNPs were dropped onto 10 mm × 10 mm Corning glass and dried in a vacuum before SEM examination. TEM images of the AuNPs and LL-37@AuNPs were captured using transmission electron microscopy (transmission electron microscopy (TEM) by a JEOL JEM 2100 (New England, USA) at an accelerating voltage of 200 kV). The microscope has a 200 kV accelerating voltage, a 0.14 nm line resolution, and a magnification of up to 1030 Kx. Up to 5 μL of the sample suspension was dropped onto a copper-coated TEM grid and the samples were allowed to dry at room temperature before being subjected to TEM imaging.

### 3.5. Biological Analysis

#### 3.5.1. Pharmacology

- Animals

The in vivo experiments were conducted on male ICR mice (25–30 g). They were purchased by the vivarium of the Institute of Neurobiology at the Bulgarian Academy of Sciences. Before the experiments, the animals were adapted for a week in standard conditions (21 ± 2 °C, light/dark regime (12/12) with a light on at 07:00 a.m.), kept in plexiglass cages, with 6–7 per cage, and tap water and animal pellets distributed ad libitum. All procedures were performed in agreement with the with the Declaration of Helsinki on the care and use of animals (DHEW publication, NHI 80-23) and the EC Directive 2010/63/EU on animal experimentation, and were approved by the Bulgarian Food Safety Authority (license number: 354/2023).

- Drugs and dosage

The nanoparticles of the peptide analogues were suspended in saline and prepared at doses of 0.25, 0.33, and 0.5 mg/kg. Before an injection, they were vortexed. The mice were infused either intracerebroventricularly (i.c.v.) (5 μsL/ventricle) as described previously [35] or injected intraperitoneally (i.p.) (0.1 mL/10 g). The referent drug phenytoin was injected i.p. at a dose of 30 mg/kg. The tests were performed 15 min (i.c.v.) or 30 min (i.p.) after drug administration.

#### 3.5.2. Tests for Anticonvulsant Activity Determination

- 6-Hz seizure test

The psychomotor seizures were assessed by stimulation (32 mA, 6 Hz for 3 s) with corneal electrodes attached to a Constant Current Shock Generator, as described earlier [34]. The stimulated mice showed various stereotyped responses, including minimal clonic seizures, a twitching of the vibrissae, blinking, or Straub-tail. The criterion for anti-seizure activity was accepted when the mouse resumed a normal position until ten min after the stimulation.

- Maximal electroshock test (MES test)

Tonic–clonic seizures were induced by electrical stimulation (50 mA, 60 Hz, 0.2 s) with corneal electrodes. The appearance of clonic seizures instead of hind limb tonic extension was an indicator of seizure suppression. Each mouse was used only once.

#### 3.5.3. Statistics

For the 6-Hz test and MES test, a Fisher’s exact test (two-tailed) was applied (SigmaPlot 11.0 version and Graph Pad Prizm Version 7.04).

## 4. Conclusions

Using a simple procedure to obtain the nanopeptide (spinorphin) aggregates in one step, the nanomaterial and peptide nanosystems based on the Rh-S@AuNP, Rh-S5@AuNP, and Rh-S6@AuNPs conjugates were developed. The gold nanoparticles used offer a promising platform for the targeted delivery of the studied spinorphin molecules to brain regions. The physicochemical properties, as well as the microstructure characteristics of the Rh-S@AuNP, Rh-S5@AuNP, and Rh-S6@AuNP conjugates, meet the requirements for use as a bioactive material. The determined values of the zeta potential of the solutions of the nanopeptide systems showed that they are high enough to consider the solutions stable and the resulting materials do not aggregate or stick together. In applications such as targeted drug delivery, achieving a high positive or negative zeta potential is important to ensure that the particles remain dispersed in the bloodstream and are less likely to form aggregates, which can affect targeting accuracy. Due to their nanoscale and other nanomaterial characteristics, the conjugates of Rh-S@AuNPs, Rh-S5@AuNPs, Rh-S6@AuNPs exhibit anticonvulsant activity commensurate with the parent compounds and good resistance. These characteristics make them a viable option for a drug delivery system. Although the three nanopeptide (spinorphin) conjugates did not show higher anticonvulsant efficacy when compared to the previously reported spinorphin peptide compounds, Rh-S, Rh-S5, and Rh-S6, respectively, the Rh-S@AuNPs showed activity that suppressed tonic–clonic seizures after a systemic administration, suggesting their potency to cross the blood–brain barrier. However, further research is needed to address the concerns related to toxicity, immune response, and regulatory approval before they can be widely used in clinical practice. Despite their biocompatibility, there are concerns about the long-term toxicity of gold nanoparticles, especially with regard to their accumulation in organs such as the liver and spleen. Translating laboratory success into clinical applications requires overcoming the challenges of scaling up production and meeting the regulatory requirements for clinical trials.

## Figures and Tables

**Table 1:** The protection and mortality rates of Rh-S and Rh-S@AuNP, administered i.p., at doses of 0.25, 0.33, and 0.5 mg/kg, respectively, for the MES test in mice.

Compound	% Protection	% Mortality
control	0	70
Rh-S		
0.25 mg/kg	0	67
0.33 mg/kg	0	67
0.5 mg/kg	0	67
Rh-S@AuNP		
0.25 mg/kg	50%	33
0.33 mg/kg	71%	0
0.5 mg/kg	86%	0

## Data Availability

Data is contained within the article.
